# Supplementary material for: Analytical Method Development and Dermal Absorption of 4-Amino-3-Nitrophenol (4A3NP), a Hair Dye Ingredient under the Oxidative or Non-Oxidative Condition
Source: Toxics. 2024 May 7;12(5):340. doi: 10.3390/toxics12050340 (PMC11125934; doi:10.3390/toxics12050340)

Figure S1. Chromatogram of 4-amino-3-nitrophenol (500 ng/mL) and 2-aminophenol (IS) in methanol (A) and ethanol (B).

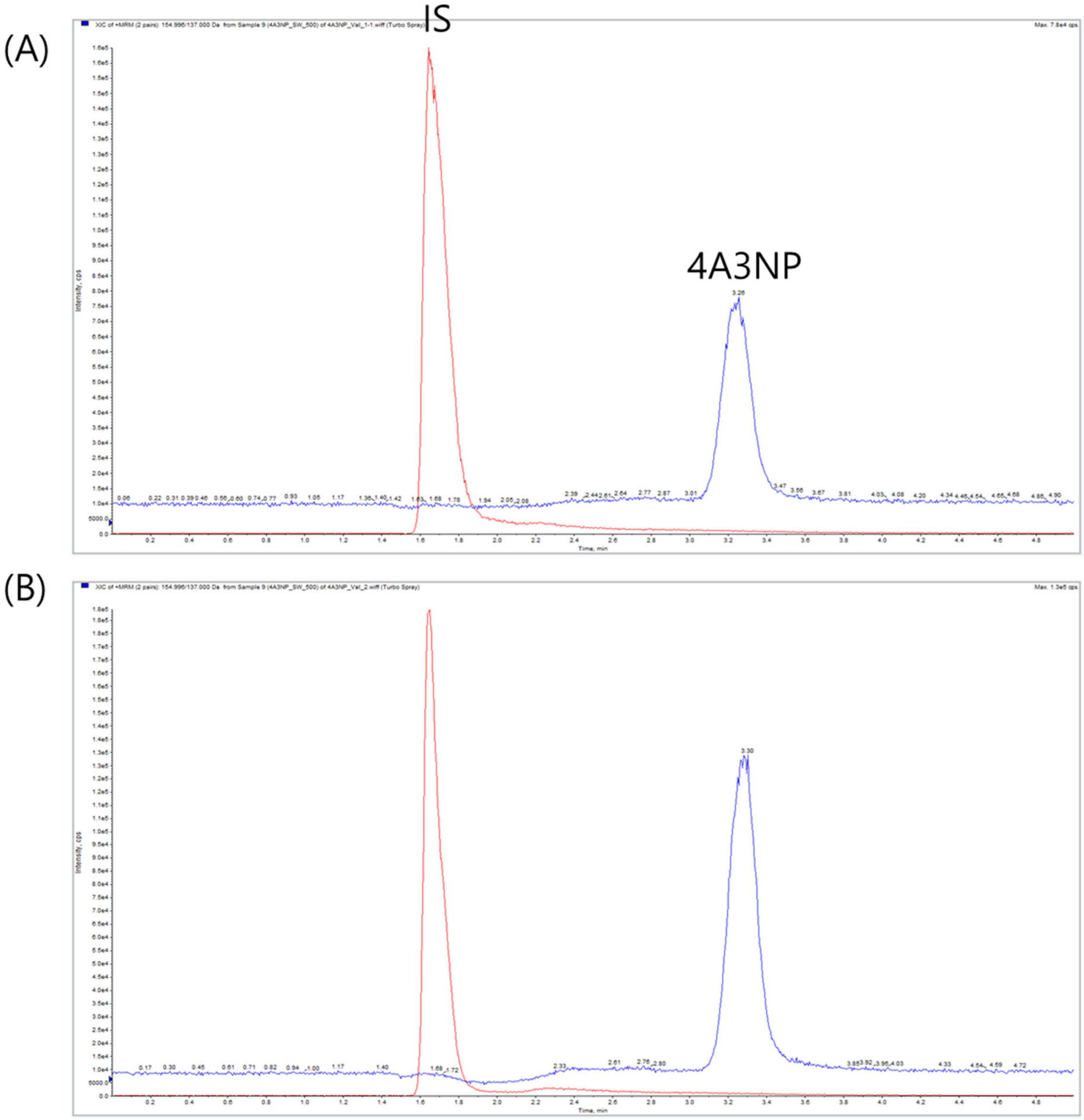

Figure S2.Calibration curves (A) and No carry-over (B)

(A) Calibration curves of 4A3NP in each matrix

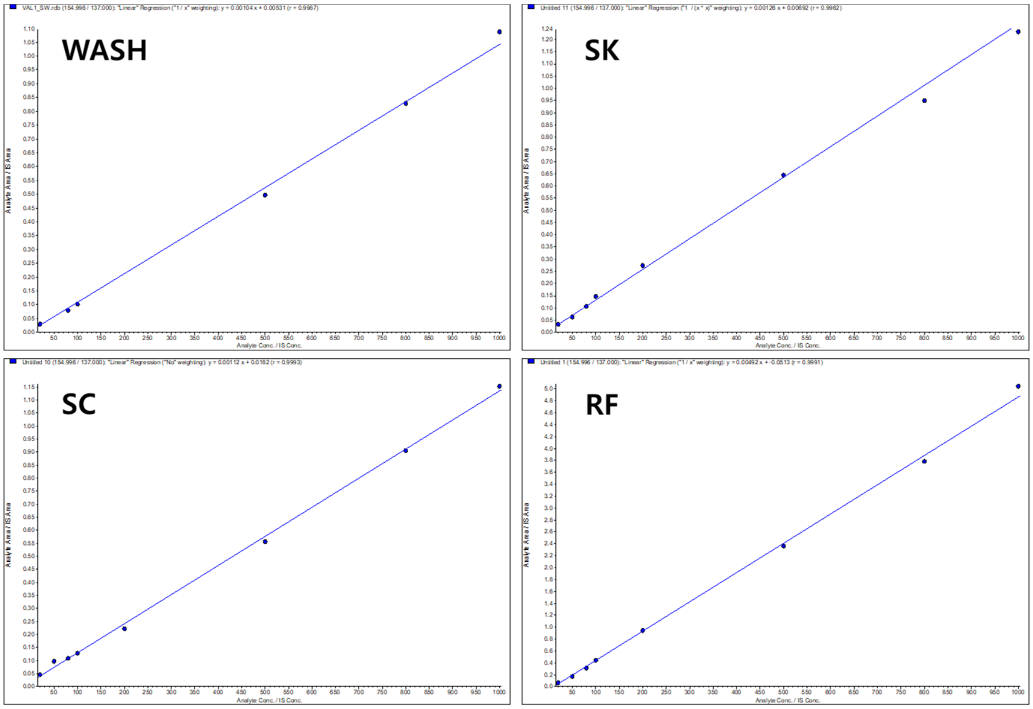

(B) No carry-over effects of 4A3NP by 750 μg/mL in each matrix

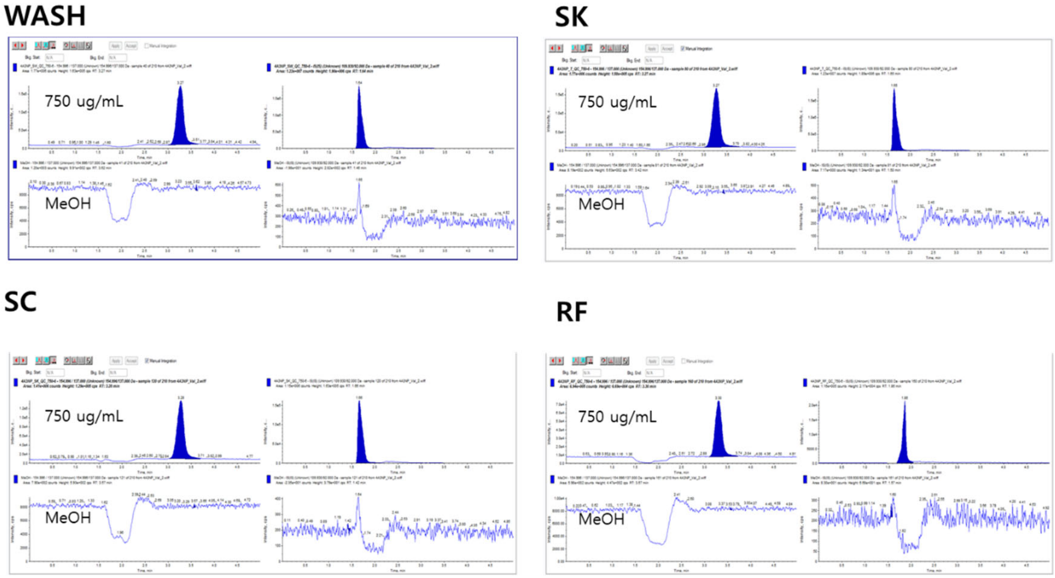

Figure S3. Chromatograms at (A) the WASH_30 min (highest concentration) and (B) SC (lowest concentration) samples.

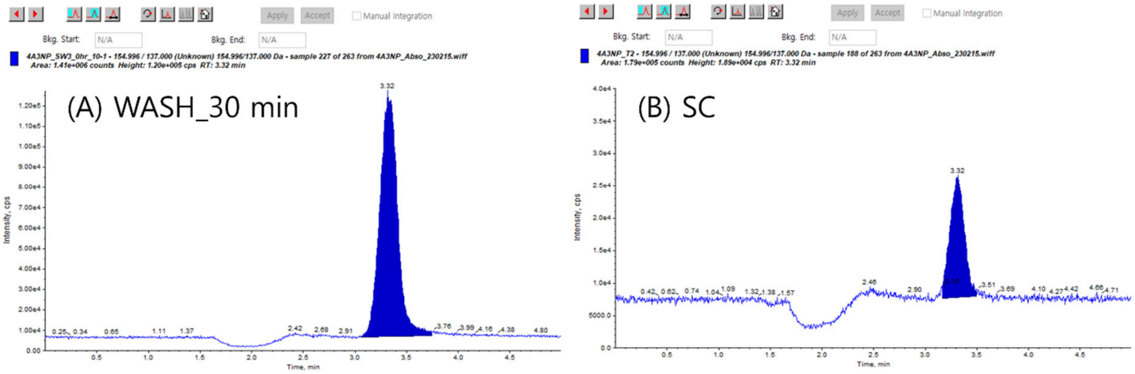

Supplement: Supplementary file 1 [file toxics-12-00340-s001.zip › toxics-2948700-supplementary.pdf]
